# Ability to Remove Na^+^ and Retain K^+^ Correlates with Salt Tolerance in Two Maize Inbred Lines Seedlings

**DOI:** 10.3389/fpls.2016.01716

**Published:** 2016-11-16

**Authors:** Yong Gao, Yi Lu, Meiqin Wu, Enxing Liang, Yan Li, Dongping Zhang, Zhitong Yin, Xiaoyun Ren, Yi Dai, Dexiang Deng, Jianmin Chen

**Affiliations:** ^1^College of Bioscience and Biotechnology, Yangzhou UniversityYangzhou, China; ^2^Jiangsu Key Laboratories of Crop Genetics and Physiology and Plant Functional Genomics of the Ministry of Education, Yangzhou UniversityYangzhou, China; ^3^State Key Laboratory of Crop Biology, College of Agronomy, Shandong Agricultural UniversityTaian, China

**Keywords:** maize, salt stress, osmotic stress, ion toxicity, physiology, transcription

## Abstract

Maize is moderately sensitive to salt stress; therefore, soil salinity is a serious threat to its production worldwide. Here, excellent salt-tolerant maize inbred line TL1317 and extremely salt-sensitive maize inbred line SL1303 were screened to understand the maize response to salt stress and its tolerance mechanisms. Relative water content, membrane stability index, stomatal conductance, chlorophyll content, maximum photochemical efficiency, photochemical efficiency, shoot and root fresh/dry weight, and proline and water soluble sugar content analyses were used to identify that the physiological effects of osmotic stress of salt stress were obvious and manifested at about 3 days after salt stress in maize. Moreover, the ion concentration of two maize inbred lines revealed that the salt-tolerant maize inbred line could maintain low Na^+^ concentration by accumulating Na^+^ in old leaves and gradually shedding them to exclude excessive Na^+^. Furthermore, the K^+^ uptake and retention abilities of roots were important in maintaining K^+^ homeostasis for salt tolerance in maize. RNA-seq and qPCR results revealed some Na^+^/H^+^ antiporter genes and Ca^2+^ transport genes were up-regulated faster and higher in TL1317 than those in SL1303. Some K^+^ transport genes were down-regulated in SL1303 but up-regulated in TL1317. RNA-seq results, along with the phenotype and physiological results, suggested that the salt-tolerant maize inbred line TL1317 possesses more rapidly and effectively responses to remove toxic Na^+^ ions and maintain K^+^ under salt stress than the salt-sensitive maize inbred line SL1303. This response should facilitate cell homoeostasis under salt stress and result in salt tolerance in TL1317.

## Introduction

Salinity is a major abiotic stress that affects plant growth and yield. More than 800 million hectares of land worldwide are affected with salt. This amount accounts for more than 6% of the world’s total land area (Zhu, 2002; Munns and Tester, 2008). However, the world demand for maize, rice, and wheat is projected to increase by 33% in 2050 (FAO). With regard to increasing world food demand and soil salinization, attention should be focused on further understanding plant response to salt stress and improving salt resistance in important crop plants (Flowers, 2004; Richter et al., 2015).

Salt resistance is a complex phenomenon, and plants manifest various adaptations at subcellular, cellular, and organ levels, such as stomatal regulation, ion homeostasis, hormonal balance, activation of the antioxidant defense system, osmotic adjustment, and maintenance of tissue water status, to grow successfully under salinity (Bartels and Sunkar, 2005; Farooq et al., 2015; Garcia-Caparros et al., 2016). The two-phase model of growth response to salt stress proposed by Munns is a significant breakthrough in understanding salt stress. According to this model, the first phase, that is, osmotic phase, begins immediately after the salt concentration around the roots increases to a threshold level (Zhu, 2001; Parida and Das, 2005; Munns and Tester, 2008). In the osmotic phase, several physiological and biochemical processes, such as photosynthesis, stomatal conductance, oxidative stress, and cellular signaling, are initially affected, and thus growth inhibition and serious tissue damage occur. Subsequently, plant growth decreases. The second phase, that is, ionic stress, affects growth in the later stage. In this phase, plants respond to salinity when ion accumulates to toxic concentrations in the tissues (Zhu, 2003; Munns and Tester, 2008). For most species, Na^+^ toxicity is a major problem in this phase of salt stress (Sumer et al., 2004; Munns and Tester, 2008). Na^+^ accumulation inhibits various important cellular processes. In addition, the homeostasis of intracellular ion concentrations is fundamental to plant growth in salinity. Many physiological studies have demonstrated that Na^+^ toxicity is caused by the ability of Na^+^ to compete for K^+^ binding sites to disrupt K^+^ homeostasis (Volkov and Amtmann, 2006; Shabala and Cuin, 2007; Hasegawa, 2013). Plant cells should maintain a low Na^+^ concentration and a concurrent high K^+^ concentration in the cytosol, where enzymes for metabolism are located, to avoid an ion homeostasis disorder under saline conditions (Zhu, 2003; Pardo et al., 2006).

Over the last two decades, the use of *Arabidopsis thaliana* and halophytes as genetic model systems advances the understanding of salt stress. Although many of these mechanisms are probably universal in most plants, their relative importance in salt tolerance may vary among species depending on the metabolic background (Flowers, 2004; Sun et al., 2010). Salt-tolerant and salt-sensitive inbred lines with relatively homozygous genotype may be ideal gene donors for improving salt tolerance in corn cultivars. Maize (*Zea mays* L.), one of the most important cereal crops, is grown under a wide spectrum of soil and climatic conditions. This crop is an important C4 plant from the Poaceae family and moderately sensitive to salt stress; nonetheless, it has wide intraspecific genetic variations for salt resistance (Farooq et al., 2015). Thus, the highly effective strategies for improving maize performance in a saline environment are urgently needed to select excellent salt-tolerant and salt-sensitive inbred lines. One population of recombinant inbred lines (RILs) or near isogenic lines (NIL) derived from a cross between the salt-tolerant and the salt-sensitive inbred lines can be used to understand the maize response to salt stress and determine the salt tolerance mechanism by QTL mapping (Flowers, 2004; Lee et al., 2004; Wang Z.F. et al., 2012; Cui et al., 2015b).

Salinity imposes osmotic stress and ionic toxicity to maize, and it shows adverse effects on growth and development. Shoot growth in maize is strongly inhibited by partially inhibiting photosynthesis or reducing the rate of transport of assimilates to growing points in the osmotic stress phase of salt stress (Yang and Lu, 2005; Qu et al., 2012). Neto found that oxidative stress may play an important role in salt-stressed maize plants through the maintenance and/or increase of the activity of antioxidant enzymes (Neto et al., 2005). De Costa observed that shoot growth of salt-tolerant maize SR03 and SR12 significantly increased in the osmotic stress phase (De Costa et al., 2007). Several studies have covered the capacity of osmotic adjustment was greater in young leaves than in mature leaves and the contribution efficiency of organic solutes to this occurrence tended to be higher in salt-tolerant maize hybrid line Arper than in salt-sensitive maize hybrid line Aristo (Hichem et al., 2009; Hajlaoui et al., 2010). In contrast to osmotic problems, a large amount of information on ion toxicity phase is available. Excessive Na^+^ accumulation is highly toxic for maize growth because of its strong interference with K^+^, which leads to disturbed stomatal regulation (Fortmeier and Schubert, 1995; Zhu, 2003; Zhang et al., 2006). Salt stress impact in terms of ionic status was more pronounced in roots than in leaves (Hichem et al., 2009; Hajlaoui et al., 2010). Schubert reported that maize inbred line Pioneer 165 uptakes low Na^+^ on the root surface to avoid Na^+^ excessive accumulation, and that inbred line Pioneer 605 maintains low Na^+^ root-to-shoot translocation to avoid Na^+^ accumulation in leaf (Schubert et al., 2009). Isla and Aragues (2010) found that certain types of maize could maintain significant amounts of Na^+^ in the stem or leaf sheaths to avoid the build-up of toxic ions in the leaf. Moreover, the high salt tolerance capability in some maize inbred lines could be closely related to their ability to maintain high K^+^/Na^+^ ratio, ion homeostasis, and membrane integrity under salt stress. (Cerda et al., 1995; Abbasi et al., 2015; Cui et al., 2015a).

Under salt stress conditions, the establishment of healthy seedlings is extremely important for subsequent maize development. Therefore, obtaining a good understanding of salt-responsive mechanisms in seedling stages is critical for improving maize salt tolerance. In the present study, an excellent salt-tolerant maize inbred line TL1317 and a extreme salt-sensitive maize inbred line SL1303 were compared in terms of their growth and physiological responses to salt stress. RNA-seq analysis was used to assess their global gene regulation through salt stress to improve the understanding of the mechanism underlying salt tolerance in maize.

## Materials and Methods

### Plant Materials and Stress Treatment

Seeds of the maize inbred lines TL1317 and SL1303 were obtained from Agricultural College of Yangzhou University. Seeds were sterilized by 10% NaClO (sodium hypochlorite) for half an hour and imbibed overnight in water. After germination in the dark at 28°C between two layers of filter paper moistened with water, the seedlings were transferred to foam boards with 10 L one-fourth concentrated nutrient concentration and grown a greenhouse with an average temperature of 28°C for 16 h at a relative humidity of about 70 ± 5%. The nutrient concentration was increased to half- and full-strength after 2 and 4 days, respectively. The full-strength nutrient solution had the following concentrations: 2.0 mM Ca(NO_3_)_2_, 1.0 mM K_2_SO_4_, 0.2 mM KH_2_PO_4_, 0.5 mM MgSO_4_, 2.0 mM CaCl_2_, 5.0 μM H_3_BO_3_, 2.0 μM MnSO_4_, 0.5 μM ZnSO_4_, 0.3 μM CuSO_4_, 0.01 μM (NH_4_)_6_Mo_7_O_24_, 200 μM Fe–EDTA. During the maize growing period, the nutrient solution was renewed every 3 days. In this study all the salt stress treatments were performed with hydroponic nutrient solution except germination assay and soil experiment.

For the germination assay, seeds were germinated in the dark at 28°C between two layers of filter paper moistened with water or 150 mmol/L NaCl solution. The number of germinated seeds was assessed 8 days after sowing on plates containing 100 seeds per genotype, with three replicates.

**FIGURE 1:**
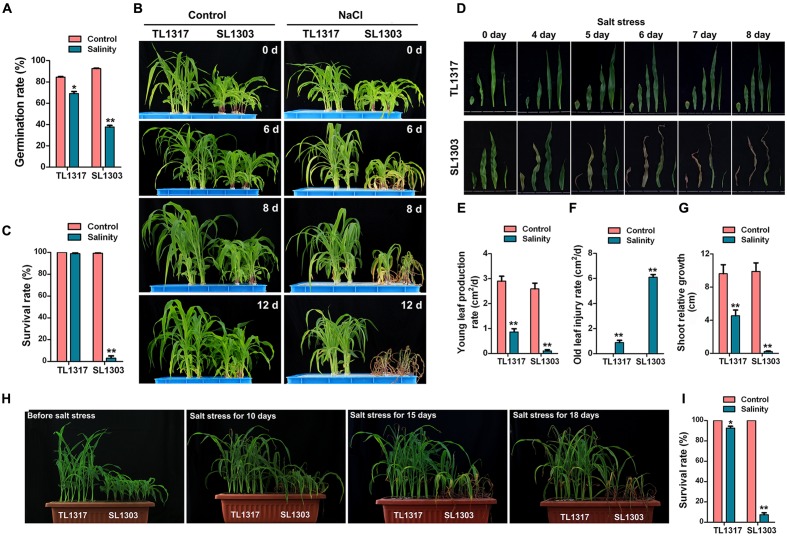
**Morphological changes in maize inbred lines TL1317 and SL1303 under salt stress.** **(A)** Comparison of germination rates between inbred lines TL1317 and SL1303 under 150 mM NaCl. **(B)** Phenotypes of inbred lines TL1317 and SL1303 seedlings grown hydroponically and treated with 150 mM NaCl. **(C)** Survival rates of inbred lines TL1317 and SL1303 seedlings grown hydroponically and treated with 150 mM NaCl for 10 days. **(D)** Leaf phenotype of inbred lines TL1317 and SL1303 (three-leaf stage) after salt stress. **(E–G)** Analysis of young leaf production rate **(E)**, old leaf injury rate **(F)**, and shoot relative growth **(G)** in inbred lines TL1317 and SL1303 after salt stress. **(H)** Phenotypes of inbred lines TL1317 and SL1303 seedlings grown in soil and treated with 250 mM NaCl. **(I)** Survival rates of inbred lines TL1317 and SL1303 seedlings grown in soil and treated with 250 mM NaCl for 20 days. Bars represent the mean (three replicates with each replicate containing 10–20 plants) ± standard deviation.

For the survival rate assay of seedlings in a hydroponic nutrient solution, three-leaf seedlings were grown in nutrient solution containing 0 and 150 mmol/L NaCl solution (**Figure 1B**). The NaCl concentration was increased gradually by 25 mM increments at daily intervals. The survival rate of seedlings was assessed after 12 days. The treatment was composed of 50 seedlings per species with three replicates. Seedlings were sampled to measure leaf area changes. Values of leaf length and leaf width were used to calculate the leaf area using the following equation: leaf area (cm^2^) = leaf length (cm) × leaf width (cm) × 0.75 (correction factor). The young leaf production rate (7 days absolute young leaf area – 0 day absolute young leaf area/7 days, cm^2^/day), old lead injury rate (7 days absolute old leaf area – 0 day old young leaf area/7 days, cm^2^/day), and shoot relative growth (7 days plant height – 0 day plant height) were calculated using 10 randomized seedlings from each species.

For the survival rate assay of seedlings in soil, seeds were planted in rectangular pots filled with the same soil mixture and watered well (**Figure 1H**). The seedlings were cultured in the same greenhouse with the same growth conditions. Three-leaf seedlings were treated with NaCl solution (250 mM). Irrigation was provided from the bottoms of the pots, and the soil was completely saturated with salt water. The survival rate of seedlings was assessed after 25 days. The treatment was composed of 30 seedlings per species with three replicates.

### Assay of Physiological Characterization

All the salt stress treatments were performed using hydroponic nutrient solution. At the three-leaf stage, the seedlings were subjected to 150 mmol/L NaCl solution.

### Relative Water Content (RWC)

The RWC was measured using the second expanded leaves of 10 plants each of TL1317 and SL1303. The leaves were cut into small pieces of one centimeter and weighted immediately (fresh weight, FW). The leaves were soaked in distilled water for 4 h to regain turgidity to constant weight, and then re-weighed as the turgor weight (TW). The leaves were finally oven-dried for 24 h at 80°C to a constant dry weight (DW). All mass measurements were made using an analytical balance with a precision of 0.001 g. RWC was measured according to the formula: RWC (%) = [(FM–DM)/(TM–DM)] × 100.

### Membrane Stability Index (MSI) Analysis

Plant MSI was determined with a conductivity meter (EL30 K; Mettler Toledo), MSI (%) = (1–initial electrical conductivity/electrical conductivity after boiling) × 100. Three-leaf seedlings were subjected to 150 mmol/L NaCl. When signs of stress began to appear on plants, seedlings were removed and immediately thoroughly rinsed with double distilled water (ddH_2_O) prior to immersion in 20 ml ddH_2_O at room temperature. After 2 h the initial conductivities of the solutions were recorded. Samples were then boiled for 30 min, cooled to room temperature and the final conductivities were measured.

### Chlorophyll Content (SPAD), Photochemical Efficiency (Fv/Fm), Photochemical Efficiency (Pn), and Stomatal Conductance Assays

The chlorophyll content (SPAD) of seedlings was estimated with a chlorophyll meter (SPAD-502, Minolta, Japan). Chlorophyll florescence was measured with a portable photosynthesis system (FMS-2, Hansatech, UK) and the maximum efficiencies of photosystem II (PSII) photochemistry, Fv/Fm = (Fm–F0)/Fm, was employed to assess changes in the primary photochemical reactions of the photosynthetic potential. One measurement of the fully expanded leaves was made for each inbred line; 20 plants from each line were used for these assays. The fully expanded leaves were selected from 20 plants grown under salt stress to measure the photosynthetic rate (Pn) and stomatal conductance using a Li-6400 Portable Photosynthesis System (LI-COR Inc. Lincoln, NE, USA).

### Biomass Assays

Shoots and roots were separated to measure FW. DW was determined after drying to consistent weight at 80°C.

### Proline Content

The determination of free proline content was performed as described by Bates et al. (1973). Tissue (250 mg) was homogenized with 10 mL of 3% sulphosalicylic acid and centrifuged at 2000 g for 5 min. Two milliliters of acid-ninhydrin and 2 mL of glacial acetic acid were added to 2 mL of supernatant, and the reaction was incubated for 1 h at 100°C. Proline was extracted with 4 mL of toluene and proline concentration was assessed by measuring A520 of the toluene phase. The proline content was calculated using standard dilutions of L-proline and expressed on an FW basis.

### Water-Soluble Carbohydrate

Total water soluble carbohydrate determination based on the phenol-sulfuric acid method (Dubois et al., 1956), involved adding 1 mL of 5% phenol solution and 5 mL of concentrated sulfuric acid to 200 μL of samples and reading the absorbance 510 nm after 20 min. Sucrose was used as standard.

### Determination of Ion Concentrations

For ion toxicity analysis, Na^+^ and K^+^ were extracted from 0.200 g samples of various tissues. Samples were ashed for 5 h in an oven at 600°C. The ashes were dissolved in 2 ml concentrated nitric acid with gentle heating. This suspension was adjusted to a volume of 50 ml with distilled water and filtered through a paper filter. Thereafter, Na^+^ and K^+^ concentrations were analyzed in the filtrate by means of atomic absorption spectrometry (PE-2100; Perkin Elmer Corporation, USA) and an inductively coupled plasma spectrometer (Optima 7300 DV; Perkin Elmer Corporation, USA).

### RNA-seq and Transcriptome Analysis

Total RNA was extracted from the roots using RNAiso Plus (Takara, Dalian, China) according to the manufacturer’s protocol. Two sets of total RNA with salt stress were prepared: one set was derived from the original RNA pool prepared from TL1317 roots at 0, 6, and 24 h time points, and the other one was obtained from the RNA pool derived from SL1303 roots at the same time points. For transcriptome sequencing and assembly, each treatment was subjected individually to digital gene expression (DGE) sequencing. Oligo(dT) beads were used to isolate poly(A) + mRNA from total RNA, and mRNAs were disrupted into short fragments using a fragmentation buffer. These short fragments were used as templates for a random hexamer primer to synthesize first-strand cDNA. The second-strand cDNA was synthesized by adding buffer, dNTP, RNase, and DNA polymerase I. The resultant short fragments were purified with a polymerase chain reaction (PCR) extraction kit and resolved with EB buffer for end reparation and addition of a poly(A) tail. Subsequently, the short fragments were connected with sequencing adapters. Following agarose gel electrophoresis, suitable fragments were selected as templates for PCR. The library was sequenced using an Illumina HiSeq^TM^ 2000 platform at the Beijing Genomics Institute^1^ (Shenzhen, China). The raw reads were stored in a FASTQ format. All clean tags were mapped to the maize reference sequence, and no more than two nucleotide mismatch was allowed. The clean tags mapped to reference sequences from multiple genes were filtered. The remaining clean tags were designed as perfect clean tags. The number of perfect clean tags for each gene was calculated and then normalized in reads per kilobase of exon model per million mapped reads (RPKM) using the method described by Mortazavi et al. (2008). For hierarchical clustering analysis, the software Cluster 3.0 was used. Functional annotation analysis of DEGs was performed by the BLAST and Blast2GO tools.

### Quantitative Real-Time PCR Analysis

Reverse transcription reactions were performed using total RNA from maize roots and leaves. First-strand cDNA was synthesized from DNase-treated total RNA with Transcriptor Reverse Transcriptase (Roche, Mannheim, IN, Germany) and oligo(dT)_18_ following the manufacturer’s instructions. Real-time PCR (qPCR) was performed using an ABI 7300 system with a SYBR Green Master Mix kit (Roche, Mannheim, IN, Germany) and specific primers. Three replicate reactions were routinely performed for each sample. Relative gene expression levels were detected using the 2^-ΔΔCT^ method (Livak and Schmittgen, 2001). Actin transcript levels were used to quantify the expression of specific genes in roots and leaves. Controls for the qPCR reactions were performed without the addition of the reverse transcriptase enzyme; these were systematically included in our experiments. All the specific primer sequences are shown in **Supplementary Table S2**.

### Statistical Analyses

Statistical analyses were performed using SPSS version 16.0 software (SPSS, Chicago, IL, USA). ANOVA was used for statistics analysis. Values represent the mean ± SD of three replicates and significant differences are indicated by asterisks in the figures.

## Results

### Salt Tolerance Differences between Maize Inbred Lines TL1317 and SL1303

#### Germination Rate of TL1317 and SL1303 Seeds

The strains used in this study were previously screened from 113 maize inbred lines. TL1317 is an excellent salt-tolerant maize inbred line, and SL1303 is a salt-sensitive maize inbred line. Salt treatment showed a clear stimulating effect on the germination of TL1317 and SL1303 seeds (**Figure 1A**). About 150 mmol/L NaCl reduced the TL1317 and SL1303 germination rate by 11.79 and 59.8%, respectively. Clearly, the inhibitory effect of salt stress on the maize inbred line SL1303 germination was more significantly different than that of the inbred line TL1317 germination.

### Salt Tolerance of Inbred Lines TL1317 and SL1303 Seedlings in Hydroponic Nutrient Solution

Inbred lines TL1317 and SL1303 seedlings were grown as described in Section “Materials and Methods.” Differences were observed at the three-leaf seedling stage (**Figure 1B**). Following exposure to NaCl stress, most inbred line TL1317 seedlings remained green and displayed continuous growth. On the contrary, inbred line SL1303 seedlings showed severe leaf wilting and yellowing after 6 days of treatment (**Figures 1B,D**). After 12 days of treatment, inbred line SL1303 seedlings were almost dead, whereas inbred lines TL1317 seedlings were still green and exhibited growth (**Figure 1B**). After 12 days of salt stress, the survival rate of inbred line TL1317 was maintained at 99.00% (**Figure 1C**). Only 3.00% of inbred line SL1303 survived after salt stress but gradually died (**Figure 1C**). This study also determined young leaf production rate, old leaf injury rate, and shoot relative growth. The young leaf production rate and shoot relative growth of inbred line SL1303 seedlings were more severely inhibited than those of inbred line TL1317 seedlings (**Figures 1E,G**). Additionally, the old leaf injury rate of inbred line SL1303 seedlings was higher than that of inbred line TL1317 seedlings (**Figure 1F**).

### Salt Tolerance of Inbred Lines TL1317 and SL1303 Seedlings in Soil

The performance of inbred lines TL1317 and SL1303 seedlings under salt stress was examined using 250 mmol/L NaCl. After 15 days of treatment, a significant difference between the two inbred lines was observed (**Figure 1H**). After 18 days of treatment, most inbred line SL1303 seedlings were wilted and dead, and inbred line TL1317 seedlings showed no visual difference. After 25 days of treatment, more than 92.00% of inbred line SL1303 seedlings were dead, and 92.59% of inbred line TL1317 seedlings survived (**Figure 1I**).

### Osmotic Effects of Salinity between Maize Inbred Lines TL1317 and SL1303 Seedlings

#### Relative Water Content and Membrane Stability Index

**FIGURE 2:**
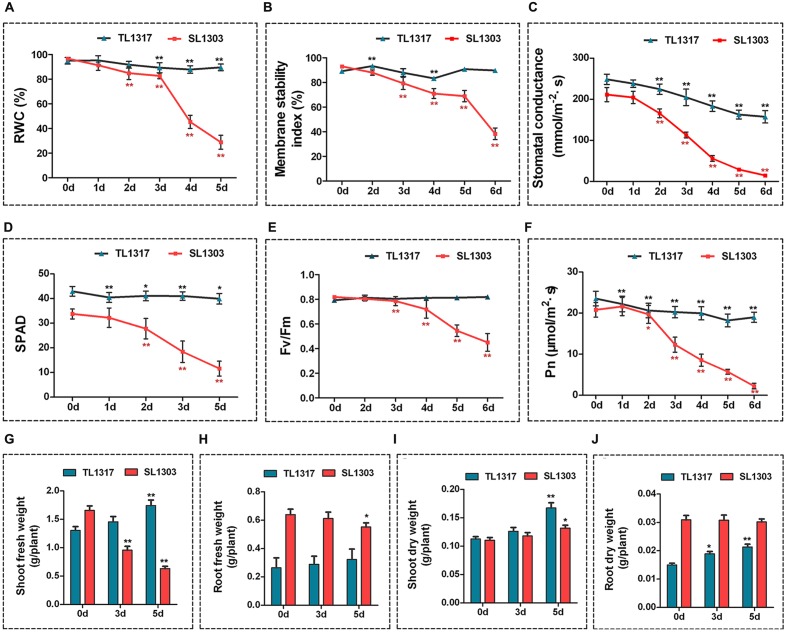
**Physiological changes in maize inbred lines TL1317 and SL1303 under salt stress.** Analysis of relative water content (RWC) **(A)**, membrane stability index (MSI) **(B)**, stomatal conductance **(C)**, chlorophyll content (SPAD) **(D)**, maximum photochemical efficiency (Fv/Fm) **(E)**, and photosynthesis rate (Pn) **(F)** in maize inbred lines TL1317 and SL1303 treated with 150 mM NaCl. **(G–J)** Changes in biomass under salt stress. Shoot fresh weight **(G)**, root fresh weight **(H)**, shoot dry weight **(I)**, and root dry weight **(J)** of inbred lines TL1317 and SL1303 with or without 150 mM NaCl treatment. Three-leaf-old seedlings were treated hydroponically and supplemented with 150 mM NaCl. Bars represent the mean (three replicates with each replicate containing 10–20 plants) ± standard deviation.

Some physiological parameters related to salt stress tolerance were assayed to ascertain physiological differences between inbred lines TL1317 and SL1303. Under normal growth conditions, the RWC and MSI of leaves were not different between inbred lines TL1317 and SL1303 seedlings (**Figures 2A,B**). After 3 days of salt treatment, the RWC and MSI of inbred line SL1303 seedlings significantly decreased, and that of inbred line TL1317 seedlings was significantly higher than that of SL1303 seedlings (**Figures 2A,B**; **Supplementary Figure S1**).

### Stomatal Conductance (Gs), Chlorophyll Content (SPAD), Maximum Photochemical Efficiency (Fv/Fm), and Photosynthesis Rate (Pn)

The stomatal conductance of inbred lines TL1317 and SL1303 seedlings was inhibited severely after 2 days of salt treatment. The reduction level of stomatal conductance was more conspicuous in the inbred line SL1303 seedlings than in the inbred line TL1317 seedlings under salt stress (**Figure 2C**). The SPAD, Fv/Fm, and Pn were measured to evaluate the photosynthetic activity of inbred lines TL1317 and SL1303. A similar tendency was observed for chlorophyll content, Fv/Fm, and Pn; the inbred line SL1303 seedlings were impaired more than the inbred line TL1317 seedlings after salt stress for 3 days (**Figures 2D–F**).

### Changes in Biomass under Salt Stress

**Figures 2G,H** show the FWs of the inbred lines TL1317 and SL1303 seedlings under salt stress. After salt stress for 5 days, the shoot and root FWs of inbred line TL1317 seedlings increased by 33.84 and 23.08% compared with those on 0 day, and those of inbred line SL1303 seedlings decreased by 62.05 and 14.06%, respectively. **Figures 2I,J** show the DWs of the inbred lines TL1317 and SL1303 seedlings under salt stress. After salt stress for 5 days, the shoot and root DWs of inbred line TL1317 seedlings increased. Moreover, the shoot DW of inbred line SL1303 seedlings increased, while the root DW of SL1303 seedlings was not significantly different (**Figures 2I,J**).

### Effects of Salt Stress on Organic Solutes

**FIGURE 3:**
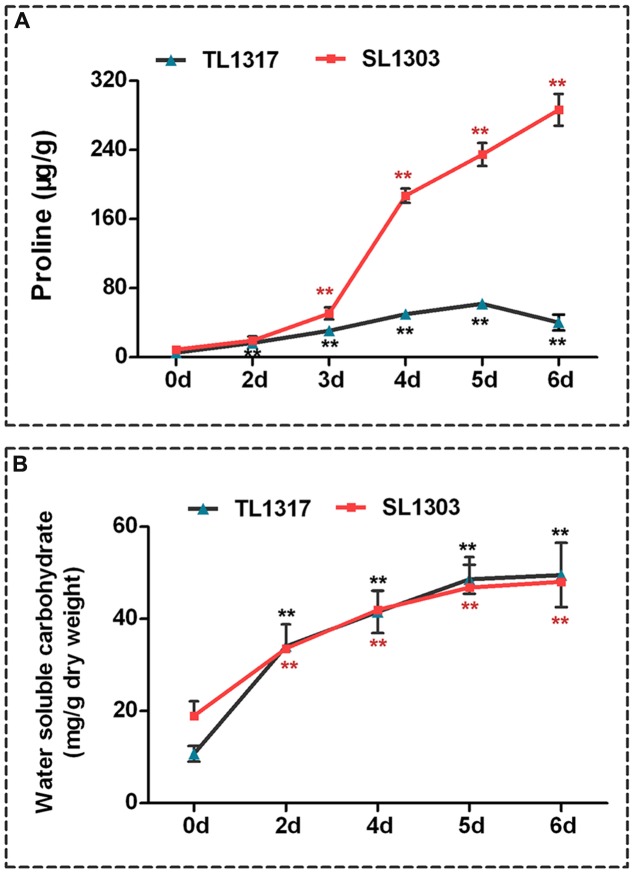
**Comparisons of proline and water-soluble carbohydrate indexes related to salt stress response.** Proline **(A)** and water-soluble carbohydrate **(B)** of maize inbred lines TL1317 and SL1303 seedlings after 150 mM NaCl treatment. Three-leaf-old seedlings were treated hydroponically and supplemented with 150 mM NaCl. Bars represent the mean (three replicates with each replicate containing 10–20 plants) ± standard deviation.

The response of proline concentration of inbred lines TL1317 seedlings was different from that of inbred line SL1303 seedlings under salt stress (**Figure 3A**). Exogenous application of NaCl caused a rapid up-regulation to the proline concentration of inbred line SL1303 seedlings at 4 days (**Figure 3A**). Afterward, the concentration continued to increase and was significantly higher than that of the control (without any stress treatment) (*P* < 0.01). At 6 days, the proline concentration of inbred line SL1303 seedlings reached a peak (32.41-fold). However, the proline concentration of inbred line TL1317 seedlings only increased to 7.6-fold compared with the control (without any stress treatment) (**Figure 3A**).

NaCl addition significantly increased the water-soluble carbohydrate contents of both inbred lines TL1317 and SL1303 seedlings. The water-soluble carbohydrate contents of both inbred lines TL1317 and SL1303 seedlings were also similar after salt stress. Nevertheless, compared with the control (without stress treatment), water-soluble carbohydrate contents were induced at higher levels in inbred line TL1317 seedlings (4.61-fold) than in the inbred line SL1303 seedlings (2.54-fold) after salt stress for 6 days (**Figure 3B**).

### Ion Effects of Salinity between Inbred Lines TL1317 and SL1303 Seedlings

**FIGURE 4:**
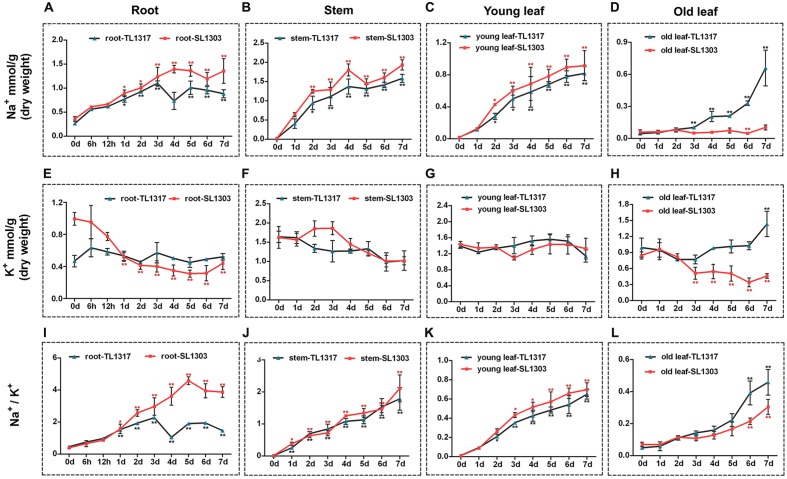
**Ion content in maize inbred lines TL1317 and SL1303 seedlings following 150 mM NaCl treatment.** Na^+^, K^+^, and Na^+^/K^+^ ratios in roots **(A,E,I)**, stems **(B,F,J)**, young leaves **(C,G,K)**, and old leaves **(D,H,L)**. Three-leaf-old seedlings were treated hydroponically and supplemented with 150 mM NaCl. Bars represent the mean (three replicates with each replicate containing 10–20 plants) ± standard deviation.

Na^+^ and K^+^ were measured after a 150 mM NaCl treatment to decipher the mechanism of ionic stress in two maize inbred lines TL1317 and SL1303. The inbred lines TL1317 and SL1303 seedlings were harvested in four parts: roots, stems, young leaves, and old leaves. After salt stress, the Na^+^ concentrations of both inbred lines TL1317 and SL1303 seedlings were significantly increased compared with those of untreated seedlings in root, stem, and young leaf. Moreover, the Na^+^ concentrations of inbred line TL1317 seedlings were significantly lower than those of inbred line SL1303 seedlings in root, stem, and young leaf. Interestingly, after salt stress for 3 days, the Na^+^ concentration of inbred line TL1317 seedlings was dramatically increased and significantly higher than that of inbred line SL1303 in old leaf. Compared with that of the control (without stress treatment), the Na^+^ concentration of inbred lines TL1317 and SL1303 increased to 14.3- and 1.7-fold after salt stress for 7 days in old leaf, respectively (**Figures 4A–D**).

Although the K^+^ concentrations of both two inbred lines in various tissues were disturbed under salt stress, the changes in K^+^ concentration in various tissues exhibited significant differences between the two inbred lines. The K^+^ concentration of inbred line SL1303 showed a dramatic reduction in root, and that of inbred line TL1317 showed little change during 7 days for salt stress in root (**Figure 4E**). The K^+^ concentration of both inbred lines TL1317 and SL1303 seedlings was reduced with salt for 7 days in stem and young leaf (**Figures 4F,G**). The K^+^ concentration of inbred line TL1317 increased under salt stress compared with that of untreated plants in old leaf, whereas that of inbred line SL1303 exhibited a reduction (**Figure 4H**).

Na^+^/K^+^ ratio is an important indicator of salt tolerance in plants. The Na^+^/K^+^ ratio of inbred line SL1303 dramatically increased with the treatment time extension in root, and inbred line TL1317 exhibited a relatively modest increase. After salt stress for 7 days, the Na^+^/K^+^ ratio of SL1303 was significantly higher than that of TL1317 in root (**Figure 4I**). The Na^+^/K^+^ ratios of both inbred lines TL1317 and SL1303 significantly increased under salt stress in stem and young leaf but showed no difference between the two lines (**Figures 4F,G**). The Na^+^/K^+^ ratios of both inbred lines TL1317 and SL1303 also had a significant increase in old leaf, and the increase in Na^+^/K^+^ ratio in inbred line TL1317 was greater than that of SL1303 in old leaf (**Figure 4H**).

### Salt-Regulated Genes in Inbred Lines TL1317 and SL1303

**FIGURE 5:**
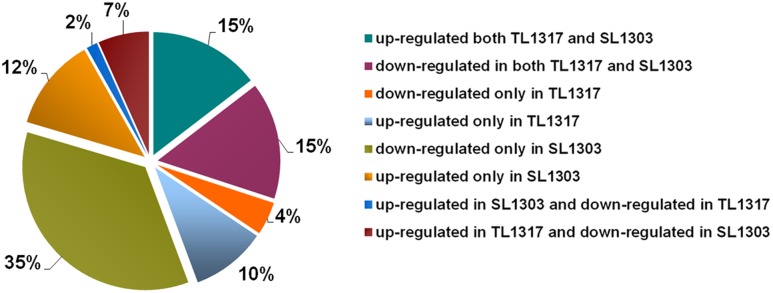
**Classifications of co-regulated and different DEGs up- or down-regulated in inbred lines TL1317 and SL1303 under salinity stress**.

To further understand the underlying molecular mechanisms, the regulation of gene expression was investigated using RNA-seq analysis. Samples of inbred lines TL1317 and SL1303 seedlings after salt stress for 0, 6, and 24 h were used for the construction of RNA-seq libraries (**Supplementary Figure S2**). RNA-seq data has be submitted to SRA database of NCBI and obtained an accession number SUB2018283. A total of 7938 successful genes were produced for at least one sample of the roots of inbred lines TL1317 and SL1303 (**Supplementary Table S1**). Of these 7938 genes, 343 significantly differentially regulated genes were classified into eight clusters according to their expression patterns. Approximately 15 and 15% of genes were up- and down-regulated in both inbred lines TL1317 and SL1303, respectively, thereby suggesting that inbred lines TL1317 and SL1303 both showed transcriptional response to salt stress to some extent. Among the 343 genes, the ratios of up- and down-regulated genes in inbred line TL1317 were 10 and 4%, respectively. The 12 and 35% genes were up- and down-regulated in SL1303 only, respectively, thus suggesting that the inbred line SL1303 is more sensitive than the inbred line TL1317 in response to salt stress. Furthermore, in the two inbred lines, only 2% of genes were up-regulated in inbred line SL1303 but down-regulated in inbred line TL1317, and 7% of genes were up-regulated in inbred line TL1317 but down-regulated in inbred line SL1303 (**Figure 5**). In the total regulated genes, the number of both up- and down-regulated genes in inbred lines SL1303 was greater than that in inbred lines TL1317 (**Figure 5**, **Supplementary Figure S2**).

By further screening and classification of genes involved in different responses to salt stress, some genes related to stress resistance and unknown genes are found. After salt stress, some stress tolerance-related genes show high expression in salt-tolerant maize inbred line TL1317. However, these genes show low expression (even down-regulated expression) in salt-sensitive maize inbred line SL1303. This variation may be related to the difference in salt-tolerance between the two inbred lines. For example, Na^+^/H^+^ transporter genes (GRMZM2G027851_T02 and GRMZM2G098494_T02), H^+^-ATP enzyme gene (GRMZM2G047875_T01), sodium/calcium exchanger gene (GRMZM2G126601_T01) and KUP protein gene (GRMZM2G020766_T01) may encode some of the important proteins that are associated with ion transport. The response of disease resistance protein RPM1 gene (GRMZM2G013170_T01), and aquaporin NIP gene (GRMZM2G082184_T02) may be associated with self-protection of the plant cell under salt stress. LRR receptor-like serine/threonine-protein kinase (GRMZM2G389948_T01, GRMZM2G025105_T01) genes are closely related to intracellular signal transduction, and they may participate in salt tolerance signal transduction. Moreover, genes related to antioxidant, transcriptional regulation, and resistance, as well as genes with unknown function, are also found with great different responses to salt stress, which may serve a function into salt-tolerant regulation (**Table 1**, **Supplementary Table S2**).

**Table 1:** Functional categories and gene expression patterns of 33 genes regulated in maize inbred lines TL1317 and SL1303 roots under salinity stress.

Gene accession No.	Expression levels (qRT-PCR)	Description	log2 (ratio) (stress/SL1303-0h)				
			
			TL1317 salt stress/SL1303-0h			SL1303 salt stress/SL1303-0h	
				
			0 h	6 h	24 h	6 h	24 h
**Sodium transport**							
GRMZM2G027S51_T02	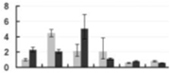	Sodium/hydrogen exchanger	-0.95	-0.72	-0.47	-0.33	-1.83
GRM2M2G063492_T01	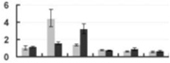	Sodium/hydrogen exchanger	-0.28	0.35	-0.11	0.17	-0.49
GRMZM2G098494_T01	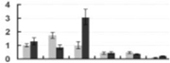	Sodium/hydrogen exchanger	0.28	0.60	0.79	0.80	-0.92
GRMZM2GQ17388_T01	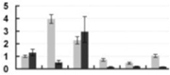	Sodium/potassium/calcium exchanger	0.41	277	1.07	-0.20	0.54
**Potassium transport**							
GRMZM2G020766_T01	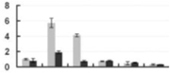	KUP system potassium uptake protein	-0.32	0.27	0.12	-0.42	-1.69
GRMZM2G126601_T01	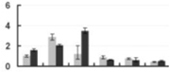	Solute carrier family 24	1.55	2.44	2.28	-0.05	-0.09
GRMZM2G351347_T01	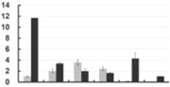	(Sodium/Potassium/calcium exchanger) potassium channel subfamily K	-4.15	2.02	3.15	4.64	4.08
GRMZM2G395267_T01	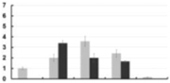	KUP system potassium uptake protein	-4.8	-4.74	-2.9	-2.44	-4.68
GRMZM2G120163_T01	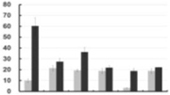	KUP system Potassium uptake protein	0.45	1.90	1.18	1.05	1.39
GRMZM2G327234_T01	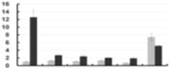	KUP system Potassium uptake protein	-2.12	-1.06	-0.90	0.39	-0.45
GRMZM2G425999_T01	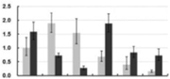	KUP system potassium uptake protein	0.28	-0.88	-0.18	-2.06	-1.13
GRMZM2G375116_T01	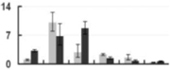	KUP system potassium uptake protein	0.08	1.73	0.87	1.83	1.10
AC234152.1_FGT002	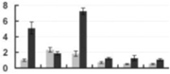	Potassium channel activity	1.93	1.63	1.51	-0.61	0.56
**Calcium transport**							
GRMZM2G352695_T01	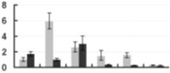	Ca2+-transporting ATPase	0.74	1.44	1.45	2.02	0,83
GRMZM2G080767_T01	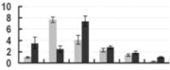	Calcium channel activity	0.17	0.69	1.47	0.46	0.71
**Hydrogen transport**							
GRMZM2G047875_T01	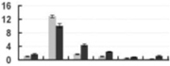	Solute: hydrogen antiporter activity	-0.07	1.01	0.01	0,25	-1.88
**Ammonium transport**							
GRMZM2G028736_T01	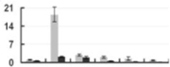	Ammonium transport	3.05	4.33	4.60	0.18	-0.86
**Anion transport**							
GRMZM2G344163_T01	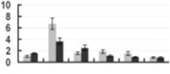	Chloride channel activity	-0.69	-0.58	-0.55	-1.09	-2.73
GRMZM2G070087_T01	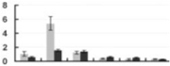	MFS transporter, inorganic phosphate transporter	2.37	-0.27	0.54	-4.94	-6.31
**Aquaporin**							
GRMZM2G082184_T02	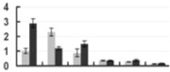	Aquaporin NIP, inorganic anion transport	3.37	3.24	3.38	0.14	0.61
**Disease resistance protein**							
GRMZM2G013170_T01	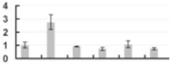	Disease resistance protein RPM1	7.13	6.98	7.25	0.31	-0.12
GRMZM2G156351_T01	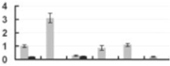	Disease resistance protein RPM1	8.63	8.48	8.91	1.58	-3.33
GRMZM2G455909_T01	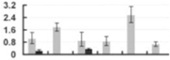	Disease resistance protein RPM1	9.04	8.68	8.72	-3.28	0.08
**Response to stress**							
GRMZM2G125032_T01	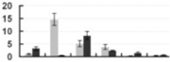	Beta-glucosidase activity	0.30	2.62	4.88	2.12	3.27
**LRR receptor-like kinase**							
GRMZM2G389948_T01	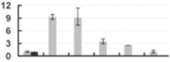	LRR receptor-like serine/threonine-protein kinase EFR	5.98	7.11	6.04	-0.01	-2.90
GRMZM2G025105_T01	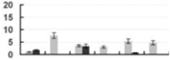	LRR receptor-like serine/threonine-protein kinase FLS2	10.43	13.39	12.50	5.46	7.55
**Others**							
GRMZM2G145518_T01	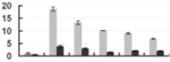	Chitinase, hydrolase activity	1.18	3.04	4.15	2.18	2.79
GRMZM2G054193_T01	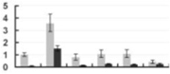	Vesicle-associated membrane protein, transport	2.51	2.57	2.45	-0.20	0.60
GRMZM2G000829T01	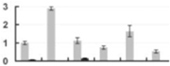	Callose synthase	7.23	7.59	7.80	-0.01	1.66
GRMZM2G149184_T01	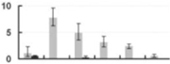	Auxin efflux carrier family	4.55	4.74	4.60	-0.01	-0.60
GRMZM2G438299_T01	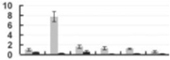	Uncharacterized protein	7.12	8.10	6.85	0.58	1.66
GRMZM2G176998_T01	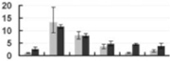	Uncharacterized protein	1.83	6.52	5.80	2.47	1.08
GRMZM2G373522_T01	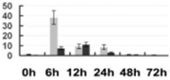	Uncharacterized protein	1.47	5.18	3.01	4.58	1.69


*Gene expression patterns of 33 genes are compared between qPCR and DGE analyses. Gene expression level is represented by the column configuration, and expression level at 0, 6, 12, 24, 48, and 72 h is shown from left to right. The gene expression level in maize inbred lines TL1317 is shown as a pale gray column, and the gene expression level in maize inbred lines SL1303 is shown as a dark grey column.*

A total of 33 candidate genes were selected for further quantitative PCR (qPCR) analyses in roots and leaves under normal and salt stress to investigate the molecular mechanisms of the responses of maize inbred lines TL1317 and SL1303 to salt stress (**Table 1**, **Supplementary Figure S3**). These gene expression levels could be induced by salt stress, but they were differently expressed in the two maize inbred lines. Although the expression level did not exactly match each other, the expression trends of all 33 genes identified through qPCR were highly consistent with the RNA-seq results. The 33 genes closely related to salt tolerance were divided into 11 categories according to different functions (**Table 1**).

The 11 categories of genes include the following: Na^+^ transport (four genes), K^+^ transport (nine genes), Ca^2+^ transport (two genes), hydrogen transport (one gene), ammonium transport (one gene), anion transport (two genes), aquaporin (one gene), disease resistance protein (three genes), LRR receptor-like kinase (two genes), stress-related (one gene), and others. Based on the structure, Na^+^ transport is mainly divided into Na^+^/H^+^ exchanger gene (GRMZM2G027851_T02 and GRMZM2G063492_T01) and Na^+^/K^+^/Ca^2+^ exchanger gene (GRMZM2G017388_T01). It is found from the comparison of two inbred lines, the expression of Na^+^/H^+^ exchanger gene and Na^+^/K^+^/Ca^2+^ exchanger gene are induced by salt stress. However, these genes are up-regulated more rapidly and have higher induced in salt-tolerance maize inbred line TL1317 than that in salt-sensitive maize inbred line SL1303. Some Na^+^/K^+^/Ca^2+^ exchanger gene (GRMZM2G351347_T01), K^+^ transport KUP family gene (GRMZM2G425999_T01, GRMZM2G120163_T01 and GRMZM2G327234_T01), and K^+^ channel activity related protein gene (AC234152.1_FGT002) showed up-regulated in salt-tolerance maize inbred line TL1317 and down-regulated in salt-sensitive maize inbred line SL1303. Meanwhile, some K^+^ transport-related genes (GRMZM2G020766_T01, GRMZM2G126601_T01 and GRMZM2G375116_T01) are up-regulated more rapidly and have higher induced in salt-tolerance maize inbred line TL1317 than that in salt-sensitive maize inbred line SL1303. Ca^2+^ transport- and signal transduction-related genes (GRMZM2G352695_T01 and GRMZM2G080767_T01, respectively) and ammonium transport related gene (GRMZM2G028736_T01) are induced in two maize inbred lines, but higher up-regulated expression was found in salt-tolerance maize inbred line TL1317. Under salt stress, the expression of aquaporin NIP gene (GRMZM2G082184_T02) is significantly increased in salt-tolerance maize inbred line TL1317 but decreased in salt-sensitive maize inbred line SL1303. After salt stress, the expression of disease resistance protein RPM1 genes (GRMZM2G013170_T01, GRMZM2G156351_T01 and GRMZM2G455909_T01) and LRR receptor-like kinase genes (GRMZM2G389948_T01 and GRMZM2G025105_T01) significantly increased in salt-tolerance maize inbred line TL1317, but decreased or no expression was found in salt-sensitive inbred line SL1303. Moreover, after salt stress, some stress-related genes and unknown genes show different expression forms in two maize inbred lines (**Table 1**).

## Discussion

As one of the most important determinants of high yield, the establishment of seedlings at the early growth stages of maize is severely affected by soil salinity. Therefore, a high germination rate and vigorous early growth under saline soils are preferred (Khayatnezhad et al., 2010). Maize is a glycophyte and moderately sensitive to salt stress; thus, soil salinity is a serious threat to its production worldwide (Farooq et al., 2015). In the present study, two different maize inbred lines, namely, salt-tolerant inbred line TL1317 and salt-sensitive inbred line SL1303, were selected from 113 maize inbred lines. The inbred line TL1317 exhibited a high germination rate and survival rate, and the ability to maintain continuous growth in both hydroponic solution with 150 mmol/L NaCl and soil with 250 mmol/L NaCl (**Figure 1**). These results showed that inbred line TL1317 exhibited better performance than other previously reported maize lines whether grown in a hydroponic solution or soil under salt stress (Qing et al., 2009; Schubert et al., 2009; Richter et al., 2015). Furthermore, we screened out the extremely salt-sensitive maize inbred line SL1303. The two maize inbred lines can support future biotechnological attempts to explore the salt tolerance mechanism and gene function in the maize.

Cell membrane stability and RWC are positively correlated with various physiological and biochemical parameter conditioning responses to salt stress (Hazel and Williams, 1990). Salt-induced effects on the RWC were used as one of the imperative water relation attributes for assessing the degree of salt tolerance in maize (Rodriguez et al., 1997) and rice (Lefevre et al., 2001). Meanwhile, salt stress immediately reduces the stomatal conductance of leaves because of perturbed water relations (Farooq et al., 2015). In the present study, The MSI, RWC and stomatal conductance of inbred line TL1317 was more stable than that of inbred line SL1303, particularly after 3 days under salt stress (**Figures 2A–C**). These results indicated that inbred line TL1317 could retain a large amount of water and high MSI, RWC, and stomatal conductance in response to salt treatment. Therefore, TL1317 exhibited better growth status than inbred line SL1303 under salt stress.

Photosynthesis, which is closely correlated with plant growth, is susceptible to salt stress (Kalaji et al., 2011). Salt stress commonly inhibits photosynthesis for two reasons, namely, stomatal and non-stomatal limitations, such as PSII (Fv/Fm), chlorophyll content, and stomatal conductance (Steduto et al., 2000). In the present study, the PSII (Fv/Fm), chlorophyll content, Pn, and stomatal conductance were less affected by salt stress in inbred line TL1317 than inbred line SL1317. In addition, the effect of osmotic stress was observed as a rapid inhibition in the rate of expansion of young leaves (Munns and Tester, 2008). The shoot and root fresh/DW showed that inbred line TL1317 maintained its growth ability, whereas inbred line SL1317 lost its growth ability under salt stress. These results suggest that inbred line TL1317 plants can maintain high photosynthetic capacity with less stomatal and non-stomatal limitations. In addition, the difference in most of the above physiological results in the two inbred lines emerged after salt stress of 3 days. Thus, the physiological effects of osmotic stress of salt stress in maize need about 3 days to manifest.

Although several roles have been attributed to the accumulation of proline upon stress, its contribution to osmotic adjustment and tolerance of plants exposed to unfavorable environmental conditions is still controversial (Ghars et al., 2008). Some data showed a high level of free proline accumulation in the salt-tolerant cultivar than in the salt-sensitive cultivar under salt stress (Misra and Gupta, 2005; Khan et al., 2009). By contrast, some studies demonstrated that the salt-sensitive cultivar accumulates more proline than salt-tolerant cultivar under salt stress (Lutts et al., 1999; Claussen, 2005). In the present study, the salt-sensitive inbred line SL1303 showed significantly higher proline accumulation than the salt-tolerant inbred line TL1317 under salt stress. Our result suggests that the excessive accumulation of proline observed in salt-sensitive inbred line SL1303 results from a loss of cell homoeostasis. Furthermore, the result indicates that the accumulation of proline itself cannot fully reflect salt tolerance, and that excessive proline accumulation may be a stress indicator.

Salt stress can break the ion homeostasis of plant cells and affect the distributions of Na^+^ and K^+^ in the cell (Niu et al., 1995). Salt stress exhibits different effects on various plant tissues (Munns and Tester, 2008). Thus, we comparatively analyzed the ion concentration in various tissues of maize inbred lines TL1317 and SL1303 seedlings and provided an opportunity to study the salt tolerance mechanism in the ionic stress of salt stress in maize. Na^+^ is the main toxic ion under salt stress in maize (Munns and Tester, 2008; Farooq et al., 2015). Na^+^ concentration of inbred line TL1317 was lower than that of inbred line SL1303 under salt stress in the root, stem, and young leaf, thereby suggesting a mechanism of Na^+^ exclusion in inbred line TL1317 seedling (**Figures 4A–C**). More importantly, in this study, the Na^+^ concentration of inbred line TL1317 showed a significant increase after salt stress compared with inbred line SL1303 in the old leaf. Combined with the phenotype results, the old leaf of salt-tolerant inbred line TL1317 gradually fell off under salt stress. This result shows that the inbred line TL1317 protects young leaves from ion toxicity by accumulating Na^+^ in old leaf after salt stress. The same mechanism, that is, the up-regulation of OsHKT1;1, OsHAK10, and OsHAK16 in rice contribution to accumulate Na^+^ in old leaves under salt stress, was observed in some other plants (Wang H. et al., 2012). However, no such data exist for salt-tolerant maize. In the present study, the Na^+^ concentration of salt-sensitive inbred line SL1303 exhibited no obvious increase in the old leaf, and the old leaf of SL1303 quickly fell off after salt stress. All of these results suggest that salt-tolerant inbred line TL1317 can exclude Na^+^ to restore ionic homeostasis through the accumulation of Na^+^ in the old leaves and the gradual shedding of these old leaves to increase the survival index of the remaining leaves.

K^+^ is an essential macronutrient for plants involved in many and important physiological processes (Hauser and Horie, 2010). Na^+^ competes with K^+^ for major binding sites in key metabolic processes in the cytoplasm because of the similarity in their physicochemical properties (Aleman et al., 2011). Furthermore, NaCl may induce K^+^ efflux from the roots of young seedlings (Cuin et al., 2008; Munns and Tester, 2008). In the present study, the K^+^ concentration was significantly different in the various tissues between inbred lines TL1317 and SL1303, specifically in roots (**Figures 4E–H**). A large number of the K^+^ efflux from the roots of salt-sensitive inbred line SL1303 after salt stress showed a strong negative correlation with salt tolerance. However, salt-tolerant inbred line TL1317 could avoid excessive Na^+^ accumulation and K^+^ efflux in root under salt stress. Therefore, the salt tolerance mechanism should inhibit Na^+^ uptake and K^+^ efflux in maize.

Na^+^/K^+^ ratio is commonly regarded as an indicator of salt tolerance in plants. A high Na^+^/K^+^ ratio under salt stress may impair the selectivity of root cell membranes and selective uptake of K^+^, thereby breaking down ion homeostasis in plants (Ashraf and Ahmad, 2000). Our results showed that the salt-tolerant inbred line TL1317 exhibited a lower Na^+^/K^+^ ratio of root and young leaf than the salt-sensitive inbred line SL1303 under NaCl treatment (**Figures 4I–L**). Combined with the MSI result, the inbred line TL1317 maintained membrane integrity and ion homeostasis more successfully than the inbred line SL1303.

A plant’s adaptation to salt stress is controlled to a high extent at the transcriptional level (Munns and Tester, 2008). The roots are the first organ that is directly affected by salt in the soil or culture solution. Therefore, we analyzed the root transcriptome of inbred lines TL1317 and SL1303 in response to salinity stress. A total of 7938 genes were analyzed using GO classification. The number of both up- and down-regulated genes in inbred line SL1303 was greater than that of inbred line TL1303; this observation, along with a significant accumulation of proline, suggested that salt-sensitive inbred line SL1303 lost its cell homoeostasis. Among these 7938 genes, 343 significantly differentially regulated genes were classified into eight clusters according to their expression patterns. The ratios of up- and down-regulated genes were 10 and 4% in inbred line TL1317, and 12 and 35% in inbred line SL1303 (**Figure 5**). More genes were down-regulated in inbred line SL1303 than in inbred line TL1317, thus suggesting that inbred line SL1303 could lose its cell homoeostasis. Moreover, a large number of both up-regulated in inbred line TL1317 and down-regulated in inbred line SL1303 suggests inbred line TL1317 may have more mechanism of salt tolerance.

Ion homeostasis is a key factor of plant survival in a saline environment (Volkov and Amtmann, 2006; Hasegawa, 2013). Na^+^/H^+^ antiporter use a proton motive force to remove toxic Na^+^ ions from the cytoplasm and maintain ion homeostasis in plant cell (Munns and Tester, 2008). Some studies demonstrated that a much greater salt stress induced Na^+^/H^+^ antiporter activity in the salt-tolerant species *Plantago maritima* than in the salt-sensitive species *Plantago media* (Staal et al., 1991). In the present study, RNA-seq and qPCR results revealed that some expressions of Na^+^/H^+^ antiporter genes (GRMZM2G027851_T02, GRMZM2G063492_T01, GRMZM2G098494_T01 and GRMZM2G017388_T01) were induced by salt stress. Nevertheless, the expression levels in inbred line TL1317 were up-regulated faster than those in inbred line SL1303 (**Table 1**). Therefore, inbred line TL1317 could activate the salt-tolerant signal pathway more rapidly and effectively than the inbred line SL1303.

Some genes involved in K^+^ transport, such as K^+^ channel, KUP family, HAK family, and KT family, help maintain low Na^+^/K^+^ concentrations and ensure ion homeostasis in plant cells (Hauser and Horie, 2010; Aleman et al., 2011). In the study, some genes related to K^+^ transport showed different expression levels in inbred lines TL1317 and SL1303 under salt stress. Some KUP family genes, K^+^ channel genes, and Na^+^/K^+^/Ca^2+^ transport genes were down-regulated in inbred line SL1303 but up-regulated in inbred line TL1317. These results suggest that the salt-tolerant maize inbred line TL1317 possesses more active responses to Na^+^ and K^+^ transport under salt stress than the salt-sensitive maize inbred line SL1303.

Ca^2+^, as a second messenger, plays an important role in the salt-tolerant signal pathway. Ca^2+^ is also a major regulator that responds to salt stress and triggers salt-tolerant mechanisms in plants (Munns and Tester, 2008). In our study, the expression of GRMZM2G352695 and GRMZM2G080767, which are genes related to Ca^2+^ transport, was induced by salt stress. The expression levels in inbred line TL1317 were also up-regulated faster and higher than those in inbred line SL1303. Therefore, inbred line TL1317 could activate the downstream Ca^2+^-dependent salt-tolerant signal pathway more rapidly and effectively than the inbred line SL1303. Moreover, leucine-rich repeat receptor-like kinases (LRR-RLK), ammonium- and anion-transport-related, aquaporin-related, and disease-resistant genes, as well as some genes with unknown functions, could be induced by salt stress. However, these genes were differently expressed in the two maize inbred lines. Therefore, these genes may be closely related to the salt-tolerant mechanism of maize. Functional studies of these genes can improve the understanding of the salt tolerance mechanism in maize.

## Author Contributions

YG and YiL carried out experiments and analyzed experimental results; MW carried out qPCR; EL took part in physiological test; YaL planted corn; DZ analyzed RNA-seq results; ZY planted corn; XR took part in ion concentration test; YD planted corn; DD assisted with planting corn; JC designed experiments and analyzed experimental results.

## Conflict of Interest Statement

The authors declare that the research was conducted in the absence of any commercial or financial relationships that could be construed as a potential conflict of interest.

## Footnotes

1 http://www.genomics.cn/index
