# Supplementary material for: Ability to Remove Na+ and Retain K+ Correlates with Salt Tolerance in Two Maize Inbred Lines Seedlings
Source: Front Plant Sci. 2016 Nov 16;7:1716. doi: 10.3389/fpls.2016.01716 (PMC5110517; doi:10.3389/fpls.2016.01716)
Supplement: Supplementary file 2 [file Table_2.DOC]

| Accession number | Forward primer | Reverse primer |
| --- | --- | --- |
| GRMZM2G027851_T02 | 5' –ACGGGGTCGGCACAGATT-3' | 5'-GTCGTGGACGCTCTGCTC'-3' |
| GRMZM2G063492_T01 | 5'- TGACTTTGATGTAGGGCAGAT-3' | 5'- CCAGGTGAGATTCAGGGAC-3' |
| GRMZM2G098494_T01 | 5'-GGTGCTCTTCGTTGGGGTG-3' | 5'-AAGGGCGACGGTGTAGGG-3' |
| GRMZM2G017388_T01 | 5'- CTCGCTCTACCTCGTCTACG -3' | 5'- TCCTGGTCGGTGTAGAACTT -3' |
| GRMZM2G020766_T01 | 5'- TGTGCGTCAAGTCCGTGC-3' | 5'- AAGTTCTCGTCGTCCTTCTGC-3' |
| GRMZM2G126601_T01 | 5'- GACATTGCTCTCCTCGCCT -3' | 5'- TGACCGAGGTTACGGATGG-3' |
| GRMZM2G351347_T01 | 5'- GCGTCGTGGAGGCAAACAAG -3' | 5'- TGGCGCACACGCAGTAGAAG -3' |
| GRMZM2G395267_T01 | 5'- CGGCGACATAGGCACATC -3' | 5'- CGGTTTCCAGGGCGTAG -3' |
| GRMZM2G120163_T01 | 5'- ACACTTCGGCACGCACAGG-3' | 5'- CCCAGTGACATCCAGCCTCC -3' |
| GRMZM2G327234_T01 | 5'-GTTACTACGGCTGGCATTTC-3' | 5'-TCATCCTCGCCCTCACC-3' |
| GRMZM2G425999_T01 | 5'-GCCTTTCCCGTCGGTCAG-3' | 5'-GAAGATGCCAGGGTAGTAGCG-3' |
| GRMZM2G375116_T01 | 5'-GGTATCTGGGCTTGAGTTGTC-3' | 5'-CAACGGATGATATTGTAGAGGC-3' |
| AC234152.1_FGT002 | 5' -GCGGCCAGGTTCCTGATATT-3' | 5' -GCTGTCCGAACTTGTGCTCTGT-3' |
| GRMZM2G352695_T01 | 5' -AGATGCTCCACTGACCGCT-3' | 5' -TTCGTGATGAACCTCCCTGT-3' |
| GRMZM2G080767_T01 | 5'- GCAGAAGATGGTGTCGGTAT -3' | 5'-AGAGCCAATCCAATCGGAGA-3' |
| GRMZM2G047875_T01 | 5'-CGCCTCACCATGTACGCAAT-3' | 5' -GAACGCCACCACCACCTG -3' |
| GRMZM2G028736_T01 | 5'-ACCTCATCTACTCCGCCTTCC-3' | 5'-ATGTGGACAACGCTGGACC-3' |
| GRMZM2G344163_T01 | 5'-GGCTTTTCGTCCCCATCAT-3' | 5'-CGGCAGCAGTAGCAGGTTGT-3' |
| GRMZM2G070087_T01 | 5'-TGGACATCGCCTTCTACAGC-3' | 5'-GACGGTGAACCAGTAGCCC-3' |
| GRMZM2G082184_T02 | 5'-TACGGCAGCGACAAGGACC-3' | 5'-TACGGCAGCGACAAGGACC-3' |
| GRMZM2G013170_T01 | 5'-GTTCTGTTAGCGACGAGGTATTG-3' | 5'-GCTTCTGTGCTGCCCTTCTT-3' |
| GRMZM2G156351_T01 | 5'-TGAAAGCAGCCGTGAGCATAC-3' | 5'-GAGGGAAGCAGGGGTCGT-3' |
| GRMZM2G455909_T01 | 5'-TCTCCCGAAAGCGATTACAAA-3' | 5'-TTGGTGGCAATAACCGACAGT-3' |
| GRMZM2G125032_T01 | 5'-AACTCGTTCCCGCCCTCC-3' | 5'-TGTCCCTGACGGTGGTGC-3' |
| GRMZM2G389948_T01 | 5'-GGAGGGCTGGGAGTTCGT-3' | 5'-TAATGCCAGGTGGTATGTTCCC-3' |
| GRMZM2G025105_T01 | 5'-AGCGTTCCGCCTGAGTTCT-3' | 5'-GGCTAAGGTCCAGCGAGTTG-3' |
| GRMZM2G145518_T01 | 5'-TACGGCGACAACTTGGACTG-3' | 5'-GAGGTGGTATTATTGGTGGTGCT-3' |
| GRMZM2G054193_T01 | 5'-GGTTTCTGAAGTCAAGGGTGTTAT-3' | 5'-CGATGATTATTCCCAGGACGAT-3' |
| GRMZM2G000829_T01 | 5'-CGTCGTGAGGCTGAGATTGC-3' | 5'-TCTTGGTTGTCTTGGCTGGAG-3' |
| GRMZM2G149184_T01 | 5'-GCTGCTGTCCTTCCACTTCA-3' | 5'-GATGGTCCAGTCCAGGTCG-3' |
| GRMZM2G438299_T01 | 5'-CGTGCTGGTGTGCGTTCTT-3' | 5'-GCCTCCAAGGTGCTCATACAAT-3' |
| GRMZM2G176998_T01 | 5'-CTGCCGCTCTTCATCCACTC-3' | 5'-TGTTCTTGCTCGCATCCTCC-3' |
| GRMZM2G373522_T01 | 5'-ACCGCACCAGCTCCAGTT-3' | 5'-TTCCTCTTGACAATTTCGCC-3' |

Supplementary Table2. Primer pairs used in quantitative real-time PCR.
