# Supplementary material for: Ability to Remove Na+ and Retain K+ Correlates with Salt Tolerance in Two Maize Inbred Lines Seedlings
Source: Front Plant Sci. 2016 Nov 16;7:1716. doi: 10.3389/fpls.2016.01716 (PMC5110517; doi:10.3389/fpls.2016.01716)
Supplement: Supplementary file 3 [file Presentation_1.PDF]

Supplementary Material

Ability to Remove Na^+ and Retain K^+ Correlates with Salt Tolerance in Two Maize Inbred Lines Seedlings

Yong Gao, Yi Lu, Meiqin Wu, Enxing Liang, Yan Li, Dongping Zhang, Zhitong Yin, Xiaoyun Ren, Yi Dai, Dexiang Deng, Jianmin Chen

*** Corresponding author::** Jianmin Chen

Email:jmchen@yzu.edu.cn

1 Supplementary Figures and Tables

1.1 Supplementary Figures

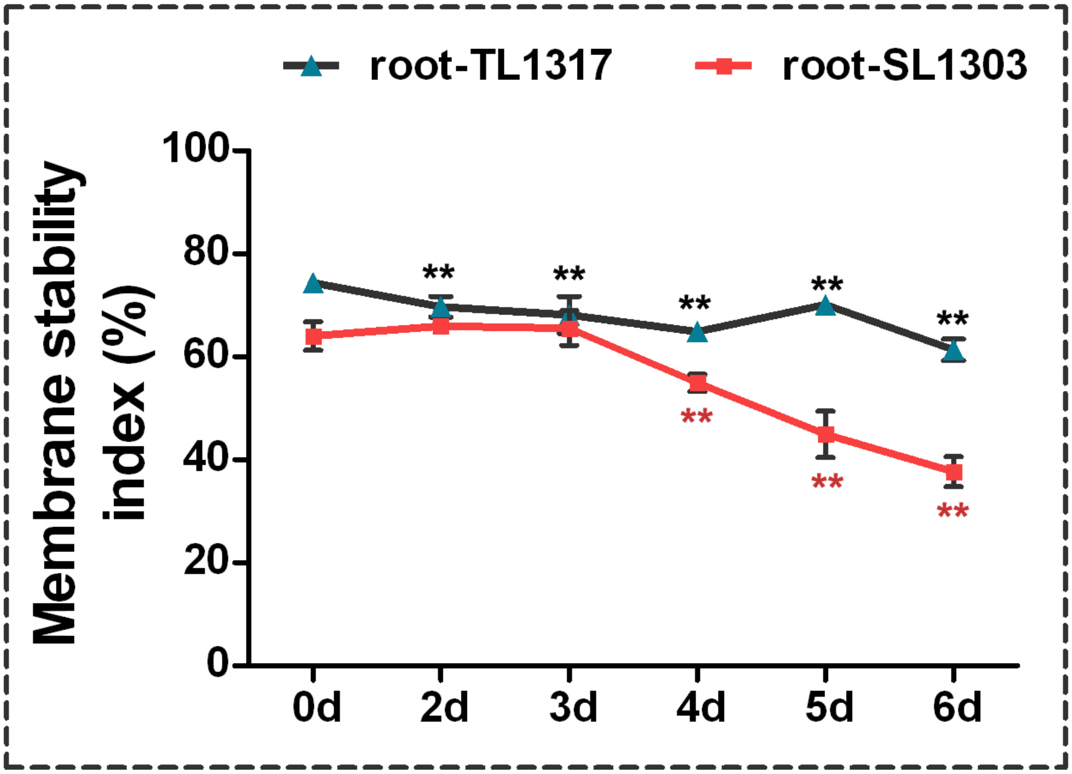

Figure S1. Membrane stability index in maize inbred lines TL1317 and SL1303 roots treated with 150 mM NaCl. Three-leaf-old seedlings were treated hydroponically and supplemented with 150 mM NaCl. Bars represent the mean (three replicates with each replicate containing 10–20 plants) ± standard deviation.

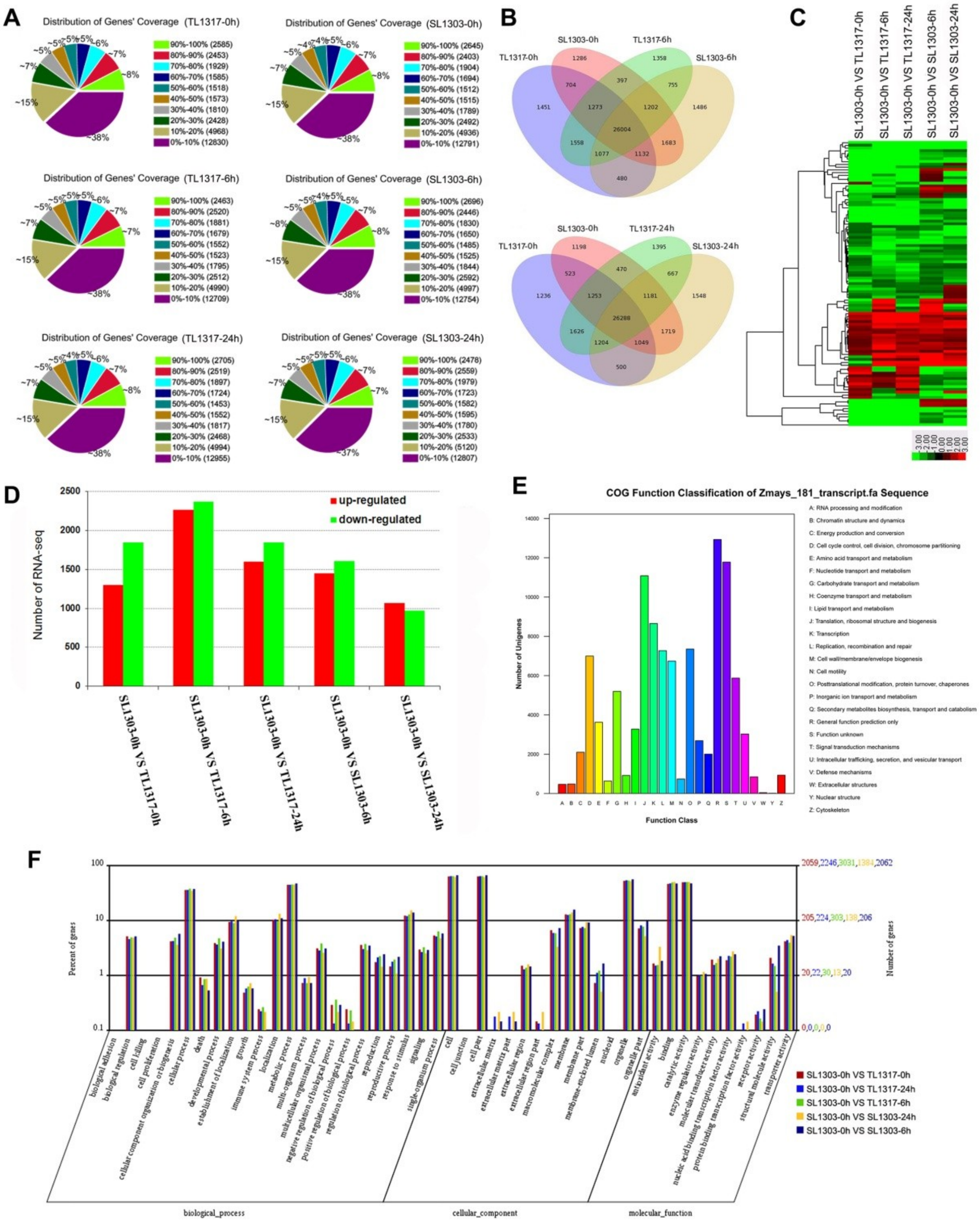

Figure S2. DEG analysis of inbred lines TL1317 and SL1303 seedlings at the three-leaf stage. (a) Distribution of gene coverage in each DGE library. (b) Distribution of genes commonly and specifically expressed in maize inbred lines TL1317 and SL1303. (c) Hierarchical cluster analysis of genes regulated using NaCl treatment. (d) Numbers of differentially expressed genes in each comparison. (e) Histogram presentation of gene COG function classification of inbred lines TL1317

and SL1303. (f) Histogram presentation of gene ontology classification of inbred lines TL1317 and SL1303.

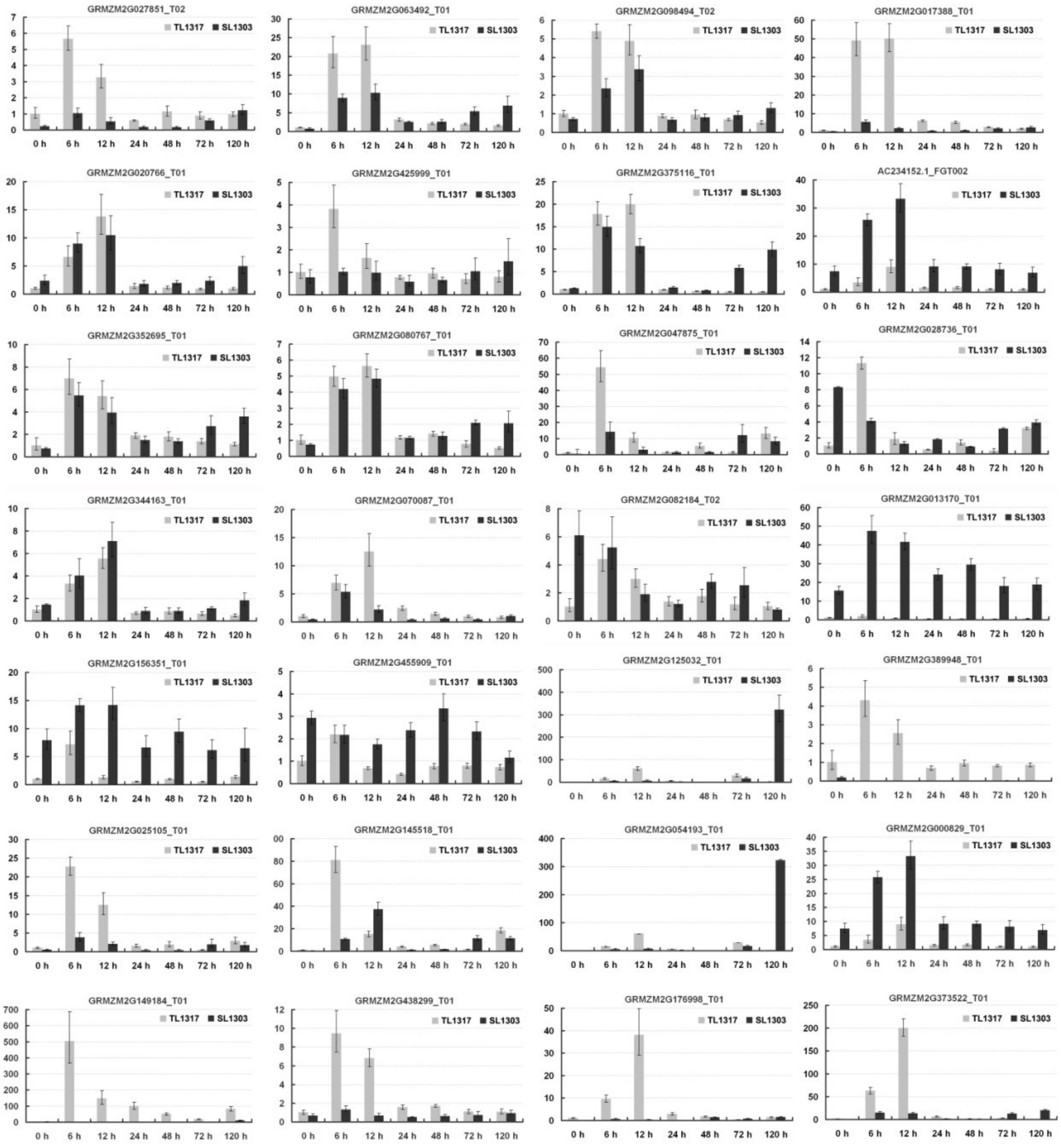

Figure S3. Gene expression patterns of 33 genes regulated in the leaves of maize inbred lines TL1317 and SL1303 under salinity stress. Gene expression level is represented by the column configuration, and expression level at 0, 6, 12, 24, 48, 72, and 120 h is shown from left to right. The gene expression level in maize inbred lines TL1317 and SL1303 is shown as a pale, gray column and dark gray column, respectively.

1.2 Supplementary Table

Table S1. List of genes commonly regulated by 150 mM NaCl in maize inbred lines TL1317 and SL1303.

This results in excel file "Table s1.xls".

Table S2. Primer pairs used in qPCR

Accession number	Forward primer	Reverse primer	Putative Function Description
GRMZM2G027851_T02	5' –ACGGGGTCGGCACAGATT-3'	5'-GTCGTGGACGCTCTGCTC'-3'	sodium/hydrogen exchanger
GRMZM2G063492_T01	5'- TGACTTTGATGTAGGGCAGAT-3'	5'- CCAGGTGAGATTCAGGGAC-3'	sodium/hydrogen exchanger
GRMZM2G098494_T01	5'-GGTGCTCTTCGTTGGGGTG-3'	5'-AAGGGCGACGGTGTAGGG-3'	sodium/hydrogen exchanger
GRMZM2G017388_T01	5'- CTCGCTCTACCTCGTCTACG -3'	5'- TCCTGGTCGGTGTAGAACTT -3'	sodium/potassium/calcium exchanger
GRMZM2G020766_T01	5'- TGTGCGTCAAGTCCGTGC-3'	5'- AAGTTCTCGTCGTCCTTCTGC-3'	KUP system potassium uptake protein
GRMZM2G126601_T01	5'- GACATTGCTCTCCTCGCCT -3'	5'- TGACCGAGGTTACGGATGG-3'	solute carrier family 24 (sodium/Potassium/calcium exchanger)
GRMZM2G351347_T01	5'- GCGTCGTGGAGGCAAACAAG -3'	5'- TGGCGCACACGCAGTAGAAG -3'	potassium channel subfamily K
GRMZM2G395267_T01	5'- CGGCGACATAGGCACATC -3'	5'- CGGTTTCCAGGGCGTAG -3'	KUP system potassium uptake protein
GRMZM2G120163_T01	5'- ACACTTCGGCACGCACAGG-3'	5'- CCCAGTGACATCCAGCCTCC -3'	KUP system Potassium uptake protein
GRMZM2G327234_T01	5'-GTTACTACGGCTGGCATTTC-3'	5'-TCATCCTCGCCCTCACC-3'	KUP system Potassium uptake protein
GRMZM2G425999_T01	5'-GCCTTTCCCGTCGGTCAG-3'	5'-GAAGATGCCAGGGTAGTAGCG-3'	KUP system potassium uptake protein
GRMZM2G375116_T01	5'-GGTATCTGGGCTTGAGTTGTC-3'	5'-CAACGGATGATATTGTAGAGGC-3'	KUP system potassium uptake protein
AC234152.1_FGT002	5' -GCGGCCAGGTTCCTGATATT-3'	5' -GCTGTCCGAACTTGTGCTCTGT-3'	potassium channel activity
GRMZM2G352695_T01	5' -AGATGCTCCACTGACCGCT-3'	5' -TTCGTGATGAACCTCCCTGT-3'	Ca^{2+}-transporting ATPase
GRMZM2G080767_T01	5'- GCAGAAGATGGTGTCGGTAT -3'	5'-AGAGCCAATCCAATCGGAGA-3'	calcium channel activity
GRMZM2G047875_T01	5'-CGCCTCACCATGTACGCAAT-3'	5' -GAACGCCACCACCACCTG -3'	solute:hydrogen antiporter activity
GRMZM2G028736_T01	5'-ACCTCATCTACTCCGCCTTCC-3'	5'-ATGTGGACAACGCTGGACC-3'	ammonium transport
GRMZM2G344163_T01	5'-GGCTTTTCGTCCCCATCAT-3'	5'-CGGCAGCAGTAGCAGGTTGT-3'	chloride channel activity
GRMZM2G070087_T01	5'-TGGACATCGCCTTCTACAGC-3'	5'-GACGGTGAACCAGTAGCCC-3'	MFS transporter, inorganic phosphate transporter
GRMZM2G082184_T02	5'-TACGGCAGCGACAAGGACC-3'	5'-TACGGCAGCGACAAGGACC-3'	aquaporin NIP, inorganic anion transport
GRMZM2G013170_T01	5'-GTTCTGTTAGCGACGAGGTATTG-3'	5'-GCTTCTGTGCTGCCCTTCTT-3'	disease resistance protein RPM1
GRMZM2G156351_T01	5'-TGAAAGCAGCCGTGAGCATAC-3'	5'-GAGGGAAGCAGGGGTCGT-3'	disease resistance protein RPM1
GRMZM2G455909_T01	5'-TCTCCCGAAAGCGATTACAAA-3'	5'-TTGGTGGCAATAACCGACAGT-3'	disease resistance protein RPM1

GRMZM2G125032_T01	5'-AACTCGTTCCCGCCCTCC-3'	5'-TGTCCCTGACGGTGGTGC-3'	beta-glucosidase activity
GRMZM2G389948_T01	5'-GGAGGGCTGGGAGTTCGT-3'	5'-TAATGCCAGGTGGTATGTTCCC-3'	LRR receptor-like serine/threonine-protein kinase EFR
GRMZM2G025105_T01	5'-AGCGTTCCGCCTGAGTTCT-3'	5'-GGCTAAGGTCCAGCGAGTTG-3'	LRR receptor-like serine/threonine-protein kinase FLS2
GRMZM2G145518_T01	5'-TACGGCGACAACTTGGACTG-3'	5'-GAGGTGGTATTATTGGTGGTGCT-3'	chitinase, hydrolase activity
GRMZM2G054193_T01	5'-GGTTTCTGAAGTCAAGGGTGTTAT-3'	5'-CGATGATTATTCCCAGGACGAT-3'	vesicle-associated membrane protein, transport
GRMZM2G000829_T01	5'-CGTCGTGAGGCTGAGATTGC-3'	5'-TCTTGGTTGTCTTGGCTGGAG-3'	callose synthase
GRMZM2G149184_T01	5'-GCTGCTGTCCTTCCACTTCA-3'	5'-GATGGTCCAGTCCAGGTCG-3'	auxin efflux carrier family
GRMZM2G438299_T01	5'-CGTGCTGGTGTGCGTTCTT-3'	5'-GCCTCCAAGGTGCTCATACAAT-3'	uncharacterized protein
GRMZM2G176998_T01	5'-CTGCCGCTCTTCATCCACTC-3'	5'-TGTTCTTGCTCGCATCCTCC-3'	uncharacterized protein
GRMZM2G373522_T01	5'-ACCGCACCAGCTCCAGTT-3'	5'-TTCCTCTTGACAATTTCGCC-3'	uncharacterized protein
